# AP-1 promotes oncogenic transcription in lung cancer cells by bridging promoter-enhancer interactions

**DOI:** 10.1038/s41417-025-00974-w

**Published:** 2025-12-03

**Authors:** Xianglong Tan, Michael Kroneberg, Fei Sun, Kevin Avelar Diaz, Alisha Flora, Michael F. Carey

**Affiliations:** Department of Biological Chemistry and Jonsson Comprehensive Cancer Center, David Geffen School of Medicine at UCLA, Los Angeles, CA USA

**Keywords:** Cancer genomics, Non-small-cell lung cancer

## Abstract

Mechanistic understanding of how transcription factors drive oncogene expression in lung cancer remains limited. Here, we utilized a cancer cell line-guided, multi-omic approach integrating promoter-capture Hi-C (pcHiC), ATAC-seq, ChIP-seq, and transcriptomics to identify epigenomic and 3D genomic alterations associated with oncogenes in non-small cell lung cancer (NSCLC) patients from The Cancer Genome Atlas (TCGA). Our cancer cell line accurately recapitulates key transcriptomic and epigenomic alterations observed in NSCLC patient samples. Comprehensive multi-omic analyses revealed aberrant activation of the bZIP family oncogenic transcription factor AP-1 in lung cancer cells. Clinically, AP-1 activation significantly correlated with patient outcomes in TCGA data, where elevated AP-1 expression levels were associated with increased mortality in lung squamous cell carcinoma patients. UMAP projections further demonstrated that AP-1-driven oncogene expression is specifically enriched in NSCLC patients exhibiting high AP-1 expression levels. Mechanistically, we observed enhanced promoter-enhancer interactions mediated by AP-1 at multiple upregulated oncogenes. Pharmacological inhibition of AP-1, either directly via AP-1 inhibitor SR11302 or indirectly through its upstream JNK pathway inhibition via SP600125, suppressed AP-1-driven oncogenic transcription and reduced promoter-enhancer looping. Our findings highlight the pivotal role of AP-1 in oncogenic transcription in NSCLC, revealing that transcription factors enhance oncogene expression by facilitating promoter-enhancer interactions.

## Introduction

Transcription dysregulation is a hallmark of cancer, driven fundamentally by the aberrant oncogene expression that facilitates uncontrolled cell growth and tumor progression [1]. During cancer progression, proto-oncogenes in early-stage cancer cells can become transcriptionally activated through genetic or non-genetic alterations. Genetic alterations involve mutations in cis-regulatory elements, such as promoters, enhancers, and insulators [2], promoting oncogenic transcription and cancer progression [3]. In contrast, non-genetic alterations pertain to trans-acting chromatin-interacting factors, including general transcription factors such as the RNA polymerase II preinitiation complex (PIC), co-activators like Mediator and EP300, chromatin regulators including histone modifiers and remodelers, as well as sequence-specific transcription factors [4]. The aberrant activity of sequence-specific transcription factors is particularly critical in driving the initiation and advancement of cancer through inappropriate activation of oncogenic transcription.

Sequence-specific transcription factors bind to specific DNA sequences known as motifs, predominantly located within promoters and enhancers, to regulate target gene expression [5]. Transcription factors also recruit co-activator complexes, such as EP300, mediator and the SWI/SNF chromatin remodeling complex, to reorganize chromatin and facilitate PIC assembly for transcription initiation [6]. Previous studies demonstrated that distinct transcription factors drive oncogenesis in a cancer-type-specific manner [7]. For instance, activator protein 1 (AP-1), one of the earliest characterized transcription factors [8], is considered to be a “double-edged sword” as it functions context-dependently either as a tumor suppressor or as an oncogenic. AP-1 transcription factors form heterodimeric complexes primarily from Jun (c*Jun*, *JunB*, *JunD*) and Fos (*Fos*, *FosB*, *Fra1*/*FOSL1* and *Fra2*/*FOSL2*) subfamily members via their bZIP domains, enabling sequence-specific DNA binding and gene regulation [9]. AP-1 is activated through multiple signaling pathways, including those involving mitogenic growth factors, inflammatory cytokines, and mutated driver oncogenes, which predominantly signal via the MAPK-JNK pathway to activate AP-1 through serine phosphorylation [9–11]. Aberrant AP-1 activation is frequently observed in cancer; indeed, *cFos* and *cJun* were initially identified as retroviral oncogenes [8]. Overexpression of *cFos* or induces osteosarcoma in mouse models [12]. Transgenic mice overexpressing *FOSL1* and *FOSL2* cause lung and epithelial tumors, respectively [13]. Clinically, *FOSL1* overexpression correlates with poor outcomes in lung adenocarcinomas [14]. Recent studies demonstrate KRAS-driven AP-1 activation recruits SWI/SNF complexes, altering chromatin accessibility to drive neoplastic transformation [15]. Additionally, AP-1 loss alters enhancer architecture and gene regulation in the context of uterine leiomyomas [16]. Besides acting independently, AP-1 can also interact with the pioneer factor FOXA1 to enhance chromatin binding in lung cancer cells [17]. Despite these findings, the detailed mechanisms by which AP-1 promotes oncogenic transcription remain unclear.

Oncogenic transcription occurs within complex three-dimensional (3D) chromatin architectures, essential for gene regulation. At different hierarchical levels, chromatin is structured into megabase-scale domains termed compartments [18], sub-megabase structures known as topologically associated domains (TADs) [19], insulated neighborhoods [20, 21] or sub-TADs [22–24], and finer-scale elements such as chromatin loops [25, 26]. Many TADs and sub-megabase structures are defined through genomic contact techniques such as ChIA-PET or Hi-ChIP at insulators [27, 28]. Among those finer-scale chromatin structures, promoter-enhancer interactions are considered as key functional interactions, which have been shown to regulate gene transcription [29]. Promoter-capture Hi-C (pcHiC), a Hi-C-derived method, allows precise mapping of these interactions [30]. We and others demonstrated that the majority of the spatiotemporal gene activation through promoter-enhancer looping occurs within insulated neighborhoods to prevent enhancers from aberrantly activating non-target genes [21, 31]. Surprisingly, the PIC influences Mediator binding but does not dramatically affect promoter–enhancer looping [32]. It remains uncertain whether sequence-specific transcription factors facilitate oncogenic expression via promoter–enhancer interactions.

In this study, we performed a comprehensive multi-omic analysis integrating promoter-capture Hi-C, ATAC-seq, ChIP-seq, and transcriptomics to investigate 3D epigenomic and transcriptional regulation of oncogenes in the NSCLC A549 cancer cell line compared with the immortalized bronchial epithelial cell line BEAS-2B (B2B). Our results were validated using data from 1041 NSCLC cancer samples and 110 normal samples from the Lung Adenocarcinoma (LUAD) and Lung Squamous Cell Carcinoma (LUSC) projects within TCGA [33–35]. Our analyses demonstrate that the cancer cell line faithfully recapitulates the key molecular characteristics of clinical NSCLC samples. We identified global increases in active promoters and enhancers but not insulators in NSCLC patient samples from TCGA. Through motif analysis, we revealed aberrant AP-1 activation in the lung cancer cell line and a subset of NSCLC patients, correlating significantly with decreased survival in LUSC patients. Mechanistically, enhanced promoter-enhancer interactions and increased AP-1 occupancy were identified at upregulated oncogenes. AP-1 inhibition, either directly via SR11302 or indirectly through JNK-MAPK pathway inhibition (SP600125), suppressed AP-1-driven oncogenic transcription, disrupted AP-1 binding, and reduced promoter-enhancer looping. Collectively, our findings highlight AP-1’s pivotal role in NSCLC progression, elucidating a mechanism whereby transcription factors enhance oncogenic expression through promoter-enhancer interactions.

## Materials and methods

### Cells

H23, A549, and B2B cells were cultured in DMEM/F-12 (Gibco) supplemented with 10% fetal bovine serum and 1% penicillin-streptomycin (Gibco) and maintained in a 37 °C humidified incubator with 5% CO_2_.

### Drug treatments for genomics

Approximately 0.5 million cells were seeded into a 6-well plate and incubated for over 12 h. SR11302 or SP600125, dissolved in DMSO, was added to the 6-well plate to achieve a final concentration of 10 µM for SR11302 or 50 µM for SP600125. After 72 h of drug treatment, the cells were harvested by trypsinization and then immediately proceeded to the subsequent protocols.

### Colony formation assay (CFA)

CFA was performed as previously described with minor modifications [36]. Briefly, 150 cells were seeded in a 6-well plate and incubated for over 12 h, after which drugs were added at indicated concentrations. The colonies were allowed to grow for 14-21 days. Following growth, the plates were washed with DPBS, and colonies were fixed and stained with 2 mL of DPBS containing 6% glutaraldehyde and 0.5% crystal violet for 1 h. The stained colonies were then rinsed with tap water and counted using Clono-Counter Software [37]. Each condition was performed in 6 biological replicates.

### mRNA-seq library preparation

Approximately 1 million cells were harvested through trypsinization. Total RNA was extracted using TRIzol. To eliminate genomic DNA contamination, the extracted RNA samples were treated with DNase I (TURBO) and subsequently purified again using TRIzol. Libraries were prepared using the KAPA mRNA HyperPrep Kit and subsequently sequenced on the Illumina NovaSeq SP/HiSeq3000 platform, achieving more than 20 million reads per library.

### ATAC-seq library preparation

ATAC-seq was performed as described by Buenrostro et al.[38]. In brief, approximately 1 million cells were harvested and lysed, after which the extracted chromatin was treated with Tn5 transposase, which had been preloaded with barcodes. Library amplification was conducted using NEBNext High-Fidelity 2× PCR Master Mix (New England Biolabs Cat #M0541), followed by size selection using a 0.7% agarose gel.

### ChIP-Seq library preparation

Approximately 13 million cells per library were collected via trypsinization. After washing with DPBS, the cells were crosslinked with 1% formaldehyde for 10 min at room temperature. Quenching was done for 5 min using 125 mM glycine, followed by two washes with cold DPBS. If not used immediately, the crosslinked cell pellets were snap-frozen and stored at −80 °C. Otherwise, the cell pellet was resuspended in 5 mL of cold Swelling Buffer (25 mM HEPES pH 7.9, 15 mM MgCl2, 10 mM KCl, 0.1% NP-40, 1× cOmplete Tablets EASYpack Protease Inhibitor Cocktail) and incubated at 4 °C for 30 min. Cells were then centrifuged at 3000 × *g* for 5 min at 4 °C and washed with 1 mL of Buffer A (50 mM HEPES pH7.9, 140 mM NaCl, 1 mM EDTA, 1% Triton X-100, 0.1% Na-deoxycholate, 0.1% SDS, 1× complete tablets EASYpack Protease Inhibitor Cocktail). The cells were resuspended in 300 µL of Buffer A and sonicated using a Qsonica Q800SR2 sonicator at 20% amplitude for 10 s “On” and 20 s “Off” cycles, totaling 3 min and 30 s of “On” time. The sonicated cells were centrifuged at 21,000 × *g* at 4 °C for 15 min. The supernatants were transferred to clean tubes and pre-cleared by incubating with Dynabeads Protein A/G for 1 h at 4 °C on a rotator. After removing the Dynabeads with a magnetic rack, 5 µL of pre-cleared supernatants were set aside as input. Antibodies were added to the remaining supernatants and incubated overnight at 4 °C on a rotator. Antibody-protein-DNA complexes were precipitated using Dynabeads Protein A/G pre-washed with Buffer A. The beads were washed twice with Buffer A, Buffer B, LiCl Buffer, and TE Buffer at room temperature. The DNA-protein conjugates were eluted with 500 µL of Elution Buffer in a shaking incubator at 65 °C and reverse crosslinked overnight at 65 °C with 140 mM NaCl and 10 µg of RNase A. Proteins were digested with 100 µg of proteinase K at 56 °C for 3 h. DNA was purified using phenol-chloroform extraction and ethanol precipitation. Libraries were prepared using KAPA HyperPrep Kits and sequenced on Illumina HiSeq 3000 or NovaSeq SP platforms.

### pcHiC library preparation

pcHiC libraries were prepared as previously described [32].

### ChIP-qPCR

The chromatin immunoprecipitation protocol was the same from the section of ChIP-Seq. After that DNA was purified using phenol-chloroform extraction and ethanol precipitation. Following qPCR was carried out using SYBR Green Master Mix and the % input was determined by 100 × 2^−ΔCt^ [39].

### Nascent RNA extraction and rt-qPCR

Chromatin-associated (nascent) RNA was isolated from 1 million of A549, H23, and B2B cells per sample using the chromatin-bound RNA extraction protocol of Schibler et al.[40]. After that, the nascent RNA from the chromatin fraction was purified with TRIzol reagent (Thermo Fisher), reverse-transcribed with SuperScript III Reverse Transcriptase (Invitrogen), and quantified by SYBR Green qPCR MasterMix (MedChemExpress). Primers targeted exonic regions of SRC and CCND1; GAPDH served as the internal control. All reactions were run in technical triplicate with no-template controls. Relative abundance was calculated by the 2^−ΔΔCt^ method, normalized to GAPDH and calibrated to the indicated control condition. Primer sequences are provided in Supplementary Table 9.

### RNA-seq data analysis

RNA-seq reads were mapped to the hg38 human reference genome with STAR (v.2.7.10a) as BAM format. Gene counts were generated by HTSeq (v.1.99.2). FPKM/RPKM values were calculated by CuffLink (v.2.2.1). Differential gene expression analysis was performed by DESeq2.

### ATAC-seq data analysis

Fastq reads from ATAC-Seq were mapped to the hg38 human reference genome using Bowtie2 (v.2.4.4). Duplicate reads were removed using a customized code. Correlations between biological replicates were calculated using deeptools (v.3.5.1). Normalization of different samples by counts per million (CPM) using samtools (v.1.13). Peak calling was carried out using MACS2 (v.2.2.7.1) *callpeak* with the—*keep-dup all* argument. Bigwig files were generated from bedGraph results from MACS2 output using bedGraphToBigWig [41]. Differential peak loci were determined by Diffbind.

### ChIP-seq data analysis

ChIP-seq sequencing reads were aligned to the hg38 human genome using Bowtie2 (v.2.4.4). Duplicate reads were removed using customized code. Correlations between biological replicates were calculated using deeptools (v.3.5.1). Subsequently, biological replicates were merged and then normalized by CPM across different cells using samtools (v.1.13). Peak calling was performed using MACS2 (v.2.2.7.1). Bigwig files were generated from bedGraph results from MACS2 output using bedGraphToBigWig. Heatmap was generated by deeptools. Differential binding analysis was performed with CPM normalized bio-replicates then followed with DiffBind (v.3.4.11).

### pcHiC data analysis

pcHiC sequencing reads were mapped to the hg38 reference genome using HiCUP (v.0.8.3). A549 and BEAS-2B (B2B) cell lines under wild-type (WT) and inhibitor treatment conditions were normalized using CPM. Pearson’s correlation, with a 1.5 kb bin cutoff, was calculated between each biological replicate for each cell line. Biological replicates were then merged using samtools (v.1.13). For global analysis, a CHiCAGO score greater than 5 was employed as a cutoff to exclude low confidence loops, and inter-chromosomal interactions were also removed. For comparing looping strength, the validated number of reads were summed and normalized by CPM between each cell line. Virtual 4C [32] was used to display looping strength.

### Motif analysis

For oncogenic motif analysis, ATAC-Seq peak files were combined with the ends of pcHiC loops from oncogenes to create a merged bed file. These bed files were further merged with transcription start sites (TSSs) of differentially expressed oncogenes to create active motif bed files. Motif analysis was then performed using HOMER (v.4.11).

### TCGA data analysis

#### Data retrieval

mRNA-Seq, somatic single nucleotide variation (SSM), copy number variation (CNV), and clinical data from the TCGA-LUAD and TCGA-LUSC projects were retrieved using TCGAbiolinks (v.2.29.6), focusing on the sample types labeled as “Primary Tumor” and “Solid Tissue Normal”.

#### ATAC-seq analysis

Bigwig files from LUAD (*n* = 44) and LUSC (*n* = 32) [35] were merged into one file each, without normalization.

#### RNA-seq analysis

**Fig. 1:**
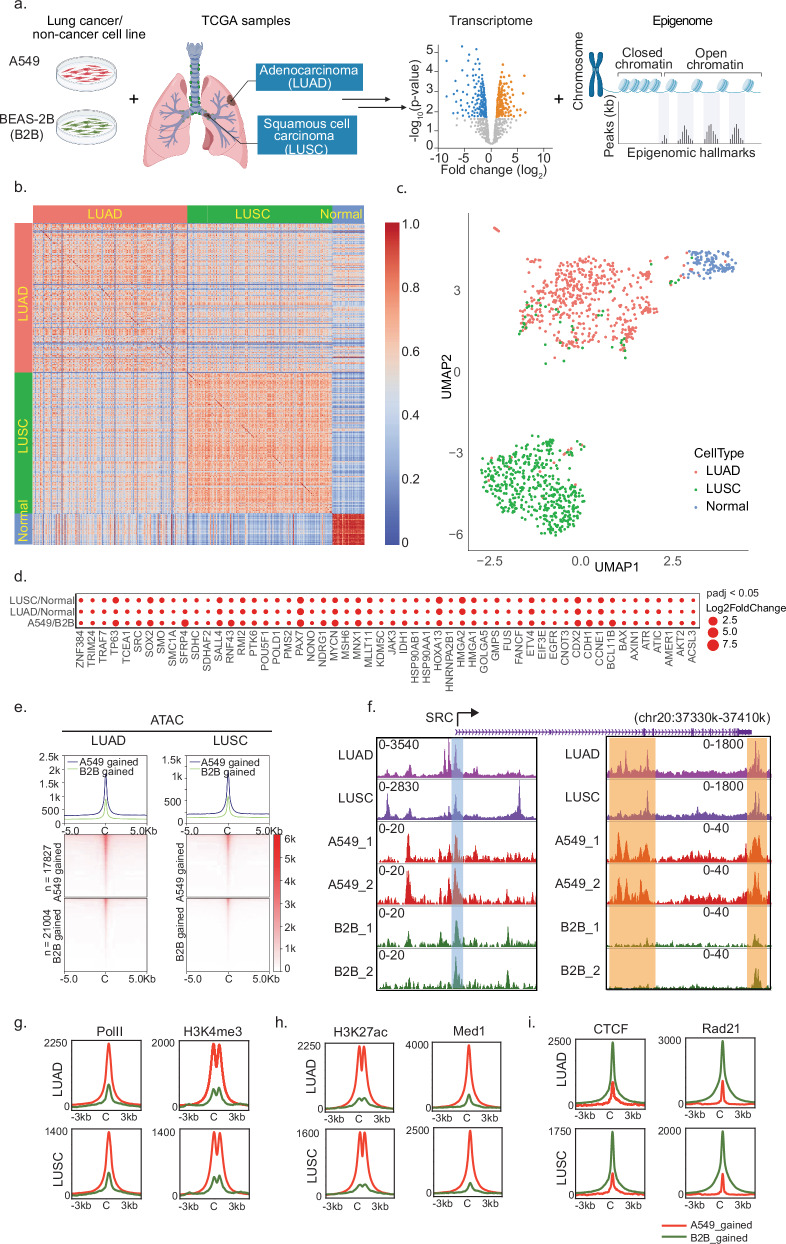
Transcriptomic and epigenomic alterations in NSCLC. **a** Schematic representation of the workflow. **b** Heatmap of Pearson’s correlation analysis for TCGA-LUAD and TCGA-LUSC transcriptomic datasets with normal peripheral tissue controls. **c** UMAP projection of normalized transcript counts between TCGA-LUAD and TCGA-LUSC cohorts. **d** Bubble plot depicting oncogenes significantly upregulated in LUAD, LUSC patient samples, and model cell lines. Significance was determined using DESeq2 with adjusted *p* values (*P*_adj_ < 0.05) after multiple testing correction. **e** Heatmap and histogram profiles of ATAC-Seq signals in combined TCGA-LUAD (*n* = 44) and TCGA-LUSC (*n* = 32) by differential binding peak loci between A549 and B2B cell line. **f** Browser track visualization centered on the upregulated oncogene SRC, featuring normalized ATAC-Seq tracks (counts per million) for A549 and B2B cells, alongside a merged track for LUADs and LUSCs. **g**–**i** Histogram profiles of ATAC-seq data from TCGA LUAD and LUSC cohorts are superimposed onto differential binding loci of ChIP-seq hallmarks from model cell lines. These include promoter hallmarks Pol II and H3K4me3 in (**g**), enhancer hallmarks H3K27ac and Med1 in (**h**), and insulator hallmark CTCF and Rad21 in (**i**). Differential binding loci were identified by DiffBind.

Pearson’s correlation analysis between RNA-seq data of TCGA cancer samples and their corresponding solid tissue normal samples was performed, as shown in Fig. 1b. This analysis and heatmap generation were carried out using a customized Python script. Differential expression analysis was executed using DESeq2 (v.1.34.0).

#### UMAP projection

Following a variance-stabilizing transformation from DESeq2, UMAP calculation was performed using the UMAP package (v.0.2.10.0).

#### Kaplan–Meier survival curve analysis

Patients were clustered based on the median value of the average Z-score of their gene sets.

## Results

### A549/B2B recapitulates key oncogenic transcriptomic and epigenomic alterations in NSCLC

To systematically analyze transcriptomic alterations of oncogenes in NSCLC, we analyzed transcriptomic datasets from 1041 NSCLC primary tumor samples and 110 normal samples within TCGA-LUAD and TCGA-LUSC. We simultaneously utilized the KRAS-G12S mutated A549 cell line and the non-cancerous bronchial epithelial cell line BEAS-2B (B2B) to measure transcriptomic and epigenomic alterations (Fig. 1a). Pearson’s correlation analysis of TCGA transcriptomes revealed strong correlation among normal samples (bottom-right heatmap), indicating minimal batch effects across projects (Fig. 1b). Additionally, LUSC samples showed high intra-group correlation and clear distinction from normal samples, while LUAD samples exhibited greater heterogeneity relative to normal samples (Fig. 1b) [42]. Uniform Manifold Approximation and Projection (UMAP), a modern dimensionality reduction approach superior to PCA for bulk transcriptomics [43], clearly separated LUAD, LUSC, and normal sample clusters (Fig. 1c). Furthermore, LUAD clusters appeared closer to normal samples, suggesting higher transcriptomic similarity. We identified 3,729 genes that are commonly upregulated and 1,977 genes that are commonly downregulated across all three comparisons: LUAD or versus peripheral normal lung tissue sample, and A549 versus B2B cells (Fig. S1a, b). To validate these findings in an independent model, we also performed the analysis using the H23 lung adenocarcinoma cell line and its matched bronchial epithelial control (BEC) from ENCODE, which revealing 2517 consistently upregulated and 1157 consistently downregulated genes across the analogous three comparisons (Fig. S1c, d). Since the A549/B2B pairs exhibited greater concordance with TCGA-derived differential expression profiles, we elected to use this model for all subsequent integrative epigenomic and 3D-genomic analyses. We subsequently analyzed transcriptomic alterations of oncogenes by merging two oncogene databases, COSMIC and OncoKB™, generating a refined set of 604 oncogenes that coexist in those two databases [44, 45] (Supplementary Table 1). Differential expression analysis indicated significant oncogenic transcriptional changes in both A549/B2B cell comparisons and TCGA patient samples (Fig. S1e–g). Notably, we identified multiple upregulated oncogenes consistently altered across LUAD/normal, LUSC/normal, and A549/B2B comparisons (Figs. 1d and S1h). To determine whether oncogene upregulation reflects increased transcription rather than mRNA stabilization, we quantified chromatin-associated nascent RNA by RT-qPCR using exon-targeting primers for SRC and CCNE1 in A549, H23, and B2B cells. Both genes exhibited higher nascent transcript levels in A549 and H23 relative to B2B cells, indicating transcriptional upregulation (Fig. S1j).

Next, we examined NSCLC-associated chromatin accessibility changes using ATAC-seq data from 44 LUAD and 32 LUSC TCGA samples, compared against our model cell lines. Heatmap of Pearson’s correlation analysis, comparing combined LUAD and LUSC patient samples with our model cells, revealed a higher correlation coefficient with the A549 cell line compared to the B2B cell line (Fig. S1j). Differential chromatin accessibility analysis revealed 17,827 open chromatin loci gained in the A549 cell line and 21,004 loci gained in the B2B cell line (Fig. S1k). The integrative analysis of LUAD and LUSC cohorts revealed increased chromatin accessibility in the loci enriched in A549 compared to B2B cells (Fig. 1e). This observation suggests alterations in the chromatin landscape observed in the NSCLC cell line are consistent with those found in NSCLC patients. For example, SRC, the first identified oncogene [46], displayed consistent transcriptional upregulation across LUAD and LUSC samples and A549 cells (Fig. 1d). ATAC-seq analysis demonstrated shared accessibility at SRC’s TSS across all samples (highlighted in blue), whereas unique open chromatin regions were observed within the gene body exclusively in cancer samples (highlighted in orange; Fig. 1f). Collectively, these results suggest that our model cell system reflects several key transcriptomic and chromatin accessibility features observed in NSCLC patient samples, supporting its utility as a model for studying oncogenic mechanisms in NSCLC.

### Epigenomic hallmark alterations in NSCLC

To investigate epigenomic features mediating oncogenic transcription in NSCLC, we performed ChIP-seq assays in A549 and B2B cells, classifying open chromatin regions as promoters, enhancers, or insulators, and integrated these data with open chromatin profiles from TCGA samples. Promoter activity was assessed using Pol II and H3K4me3 ChIP-seq [34]. Heatmap analysis of RPKM-ranked TSSs revealed increased enrichment of these promoter markers at actively transcribed genes in A549 cells compared to B2B (Fig. S1l). Differential binding analysis further confirmed enhanced chromatin accessibility at Pol II and H3K4me3 sites in LUAD and LUSC patient samples corresponding to loci enriched in A549 cells (Fig. 1g), indicating increased promoter activity in NSCLC.

We next analyzed active enhancer regions marked by Mediator subunit Med1 and histone H3K27ac modifications. While Med1 intensity at TSSs remained comparable between A549 and B2B cells, H3K27ac signals increased more than threefold in A549 cells, mirroring the promoter patterns observed with Pol II and H3K4me3 (Fig. S1m). Differential binding analysis integrating LUAD and LUSC ATAC-seq data similarly showed increased binding at enhancer-associated loci enriched in A549 cells (Fig. 1h), suggesting elevated enhancer activity in NSCLC.

Lastly, we investigated insulator changes by performing ChIP-seq for insulator proteins CTCF and Rad21 (a cohesin subunit). CTCF and Rad21 exhibited comparable binding intensities between A549 and B2B cells (Fig. S1n). However, differential chromatin accessibility analysis revealed higher enrichment at insulator-associated loci preferentially enriched in B2B rather than A549 cells (Fig. 1i), implying reduced insulator activity in NSCLC patient samples. Together, these results demonstrate a significant gain of active promoter and enhancer epigenomic features accompanied by reduced insulator hallmarks in NSCLC.

### AP-1 is aberrantly activated in a subset of NSCLC patients

**Fig. 2:**
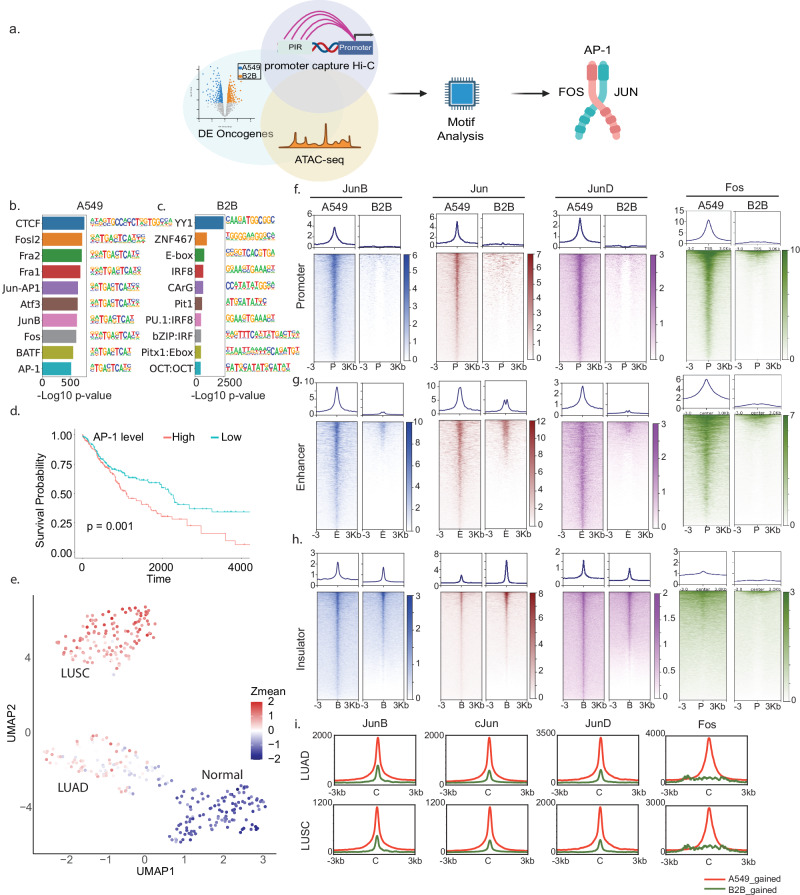
Transcription factor AP-1 is aberrantly activated in NSCLC. **a** Schematic workflow for multi-omics motif analysis in A549 and B2B cells: initially, pcHiC regions are merged with ATAC-seq chromatin accessibility in each cell line, forming merged region sets. For promoter-promoter/enhancer looping elements, these sets are further merged with significantly differentially expressed genes and subsequently with H3K27ac ChIP-Seq signals. For promoter-insulator looping elements, the merged sets are combined with CTCF and Rad21 colocalization sites. Finally, motif analysis is conducted using HOMER, which reveals the AP-1 family transcription factor. Top 10 scoring motifs in A549 (**b**) and B2B (**c**) cells. **d** Kaplan–Meier survival curves depicting patients with varying levels of AP-1 expression intensity in TCGA-LUSC cohorts. **e** UMAP projection of quantile 4 (Q4) samples from Fig. S2f alongside normal tissue samples, colored by the average Z-score of AP-1 subunit transcription levels. Heatmaps and histogram profiles demonstrate the distribution of the AP-1 family transcription factors JunB, Jun, JunD, and Fos across promoter (**f**), enhancer (**g**), and insulator (**h**) elements in A549 and B2B cells. **i** Histogram profiles of ATAC-seq data from TCGA LUAD and LUSC cohorts are superimposed onto differential binding loci of the AP-1 transcription factor from ChIP-seq analyses in model cell lines.

To identify transcription factors driving altered oncogenic expression in NSCLC, we developed a multi-omic motif analysis pipeline (Fig. 2a). Initially, we designed pcHiC probes targeting a combined list of 604 oncogenes from COSMIC and OncoKB databases (Supplementary Table 1), along with the top ~200 highly expressed genes in A549 and B2B cells (ranked by RPKM), yielding 1074 promoter-targeting probes (Supplementary Table 2 and Fig. S2a). This targeted approach enabled us to investigate most of the 3D genomic patterns of oncogenes and pinpoint critical changes in highly expressed genes between these two cell types. Additionally, focusing on a subset of approximately 5% of human genome genes enabled us to identify specific target genes at a higher resolution [47, 48]. By using 4-cutter restriction enzymes, we achieved a sequencing depth of over 500 million reads combined, yielding a final resolution of 1.5 kb. This analysis identified 15,271 valid chromatin loop loci in A549 cells and 11,759 in B2B cells (CHiCAGO score >5) (Fig. S2b, c).

Subsequently, we narrowed down our oncogenes to those differentially expressed in the A549 and B2B cells. By integrating pcHiC loops from these genes with corresponding ATAC-seq peaks, we constructed consolidated genomic locations for motif analysis using HOMER (Fig. 2a). Strikingly, 9 of the top 10 motifs uniquely enriched in A549 cells belonged to the AP-1 transcription factor family (Fig. 2b, c). To evaluate the clinical relevance of AP-1 activation, we examined the transcription levels of seven major AP-1 subunits in NSCLC patient samples from TCGA. Patients were clustered into high and low AP-1 expression groups based on the median expression level. Kaplan–Meier survival analysis revealed that LUSC patients with high AP-1 expression exhibited significantly lower survival rates (Fig. 2d), whereas no such correlation was observed in LUAD patients (Fig. S2d). To further explore this clinical associations, we subdivided the TCGA cohort samples into four quantiles (Q1-Q4) based on the mean transcriptional intensity of the seven major subunits of AP-1 family transcription factors (Fig. S2e). UMAP projection of Q4 samples (highest AP-1 expression) revealed distinct clustering of LUSC versus LUAD cohorts, both of which separated from healthy lung controls (Fig. S2f). We then generated a UMAP colored by the average Z-scores of the upregulated oncogene list (Fig. 1d). This analysis showed stronger upregulation of these oncogenes in LUSC patients compared to LUAD patients (Fig. 2e). Together, these findings suggest that aberrant activation of AP-1 in LUSC may drive oncogenic transcription programs that contribute to poor patient survival.

### AP-1 is enriched at promoters and enhancers of actively transcribed genes in NSCLC

As described above, AP-1 family transcription factors are formed through heterodimerization between a Fos family member and a Jun Family member [9]. To systematically analyze the chromatin elements bound by AP-1, we performed ChIP-Seq on all Jun family subunits (*JunB*, *cJun*, and *JunD*) and a Fos family member *cFos/Fos*, in A549 and B2B cells. We merged AP-1 ChIP-Seq signals onto the active promoters, enhancers, and insulators. All of those tested AP-1 subunits showed significantly stronger binding at active promoters and enhancers in A549 cells compared to B2B cells (Fig. 2f, g), consistent with their role in promoting oncogenic transcription in NSCLC. In contrast, binding patterns at insulator regions varied. *JunB* and *JunD* exhibited increased binding at insulators in A549 cells, *cJun* showed decreased binding compared to B2B cells, while *Fos* exhibited minimal binding at insulator elements (Fig. 2h). To orthogonally validate the ChIP-Seq profiles, we also performed *Fos* ChIP-qPCR at two representative AP-1 occupied loci, the promoter region of HSP90AB1 and the enhancer region of SRC, in A549, H23, and B2B cells. Percent-input normalized *Fos* occupancy was markedly higher in both cancer cell lines than non-cancerous cell line at each locus, confirming preferential AP-1 enrichment at active promoters and enhancers in lung cancer cells (Fig. S3f). These distinct binding patterns suggest functional divergence among AP-1 subunits at regulatory elements.

To further validate whether AP-1 binds oncogenic promoters in lung cancer cells, we merged AP-1 peaks at the list of 604 oncogenic promoter regions. Unlike ATAC-Seq signals, which displayed similar binding intensity between A549 and B2B cells, all AP-1 family transcription factors exhibited significantly enriched binding at oncogenic promoters in A549 cells than B2B cells (Fig. S2h–l). These results further support the association of AP-1 correlates with oncogenic transcription in lung cancer.

To assess clinical relevance, we performed a differential analysis of AP-1 family subunit binding sites using merged LUAD and LUSC ATAC-seq data from TCGA. The results showed enhanced chromatin accessibility at AP-1 binding sites enriched in A549 cells, indicating that these regions are also accessible in NSCLC patient tumors (Fig. 2i). This observation suggests that AP-1 may bind to those open chromatin regions in NSCLC patients. Genome browser tracks further highlighted two instances of upregulated oncogenes, HSP90AB1 and SDHAF2 (Fig. 1d), in A549 cells compared to B2B cells at TSS regions (Fig. S2g). Both upregulated oncogenes exhibited elevated signals for Pol II, Med1, and AP-1 at their TSSs, along with reduced Rad21 occupancy, suggesting enhanced transcriptional activity and decreased insulation at these loci in cancer cells (Fig. S2g). Collectively, these findings indicate that AP-1 subunits are preferentially enriched at active promoters and enhancers in NSCLC, correlating with increased oncogenic transcription and altered chromatin accessibility in both cell models and patient samples.

### AP-1 correlates oncogenic transcription via aberrant promoter-enhancer looping

**Fig. 3:**
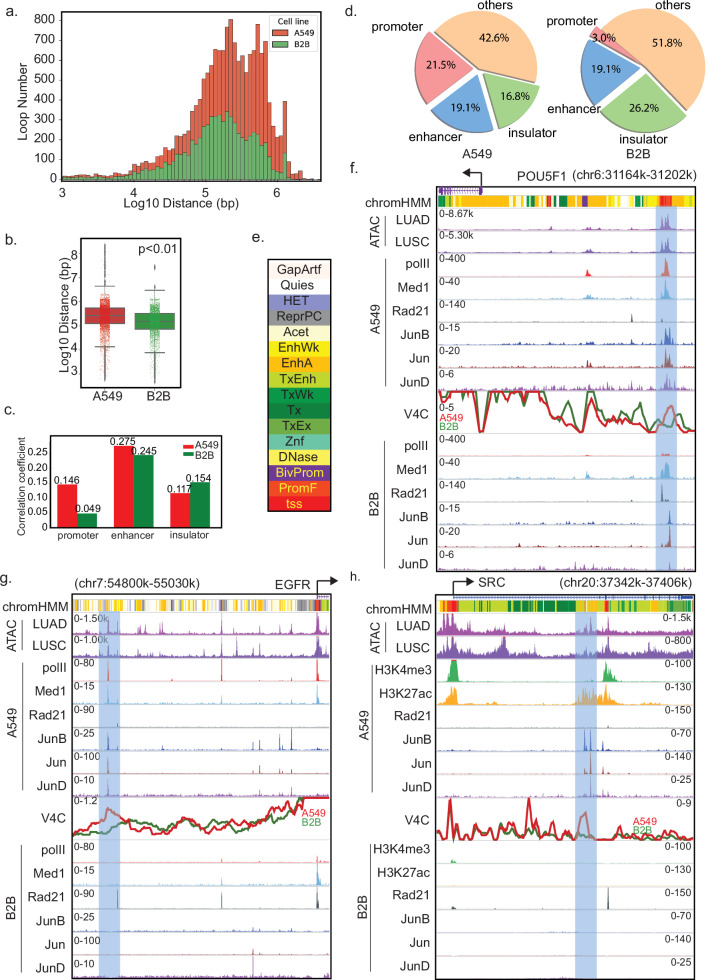
Alterations in promoter looping within oncogenes and highly transcribed genes. **a** Distribution of distance and interaction intensity between promoters and promoter-interacting regions (PIRs) for the selected set of oncogenes in A549 and B2B cells. **b** Box plot illustrating promoter looping distances in Log10 scale for the selected set of oncogenes in A549 and B2B cells. **c** Boxplot displaying Spearman’s correlation between the transcription intensity of probed genes and their promoter looping intensity to the corresponding cis-control elements in A549 and B2B cells. **d** Pie chart displaying the distribution of promoter-AP1 looping to promoters, enhancers, and insulator elements in A549 and B2B cells. **e** ChromHMM states annotation: (tss) transcription start site, (PromF) flanking promoter state, (BivProm) bivalent promoter state, (DNase) DNase sensitivity, (Znf) zinc-finger gene associated state, (TxEx) exon associated transcription state, (Tx) transcription state, (TxWk) weak transcription state, (TxEnh) transcribed enhancer state, (EnhA) active enhancer state, (EnhWk) weak enhancer state, (Acet) acetylations state, (ReprPC) polycomb repressed state, (HET) heterochromatin state, (Quies) quiescent state, (GapArtf) assembly gaps and artifacts state. Browser track showcases the upregulated oncogenes POU5F1 (**f**), EGFR (**g**), and SRC (**h**).

To further explore whether AP-1 enrichment at active promoters and enhancers in NSCLC leads to increased promoter-enhancer interactions, we conducted an integrated analysis focused on oncogenes. Initially, from our pcHiC analysis, we identified 9585 high-confidence promoter loops in the A549 cell line and 6822 in the B2B cells focused solely on oncogenes. Global oncogene loop analysis revealed an increase in both the number and distance of loops in A549 cells compared to B2B cells (Fig. 3a, b). To quantify how 3D chromatin architecture correlates with transcriptional output, we performed Spearman correlation analyses between pcHiC-delivered loop strengths and RNA-seq expression levels in both A549 and B2B cells (Fig. 3c). Promoter-enhancer contacts showed the strongest positive correlation with gene expression (*ρ* = 0.275 in A549; *ρ* = 0.245 in B2B), indicating that enhancer-promoter looping underlies oncogene activation. In contrast, promoter-promoter loops correlated only weakly in A549 (*ρ* = 0.146) and were not significantly associated with expression in B2B.

Next, we assessed AP-1’s role in mediating these interactions by selecting loops anchored at AP-1 ChIP-seq peaks and tallying their types (Fig. 3d). In A549 cells, AP-1-associated loops were relatively evenly distributed: 21.5% promoter–promoter, 19.1% enhancer–promoter, and 16.8% insulating. By comparison, B2B cells exhibited a dramatic reduction in promoter–promoter loops (3.0%), maintained a comparable level of enhancer–promoter loops (19.1%), and showed an increase in insulator-related loops (26.2%), highlighting the cancer-specific remodeling of chromatin topology by AP-1.

During our integrated analysis, we observed several specific oncogenes that highlighted both the predominant trends and interesting deviations. We utilized ChIP-Seq tracks for Pol II, Med1, and Rad21 as markers for promoters, enhancers, and insulators, respectively. For open chromatin regions, we employed ATAC-Seq data superimposed from TCGA-LUAD and TCGA-LUSC cohort samples. To showcase the pcHiC signal for each individual oncogene of interest, we utilized Virtual 4 C, a method for visualizing chromosomal interactions focused on a specific promoter [32]. Additionally, we incorporated a universal human genome ChromHMM dataset, which includes 16 chromatin states based on the similarity of chromatin hallmarks in over one thousand human epigenome datasets [49], to annotate the different universal chromatin elements (Fig. 3e). For the oncogene POU5F1, which encodes for the Oct4 Yamanaka factor, its transcription level increased in the A549 cell line as well as in TCGA cancer samples (Fig. 3f blue track). We identified a distant enhancer (highlighted in light blue) located approximately 30 kb upstream of the POU5F1 promoter loci that exhibits significant enrichment of Pol II and Med1 signals in A549 cells. This enhancer location is also marked as “active enhancer state (EnhA)” in the ChromHMM track. Interestingly, this enhancer coincides with an increase in promoter looping signals in A549 compared to B2B cells, as demonstrated by Virtual 4 C analysis. AP-1 is enriched on this enhancer in A549 compared to B2B cells. Moreover, the ATAC-Seq data from both LUAD and LUSC cohorts display enriched signals at this enhancer (Fig. 3f). This observation suggests that the enhancer is hijacked by the POU5F1 promoter, possibly through enhanced promoter-enhancer interaction facilitated by AP-1 transcription factors, leading to oncogenic transcription. Similar promoter-enhancer looping events, colocalized with aberrantly activated AP-1, were observed at approximately 20 kb and 190 kb upstream of the TSS of EGFR (Fig. 3g), and about 10 kb upstream and 40 kb downstream of the TSS of SRC (Fig. 3h), highlighting two additional oncogenes upregulated in NSCLC. To further quantify the prevalence of promoter-enhancer loop remodeling across our full oncogene cohort, we first filtered promoter-enhancer interactions to those overlapping active enhancer regions (co-localized by H3K27ac and Med1 peaks). For each oncogene, we calculated the mean pcHiC read count per promoter-enhancer interaction in A549 and B2B. We defined (∆counts = A549_count – B2B_count) as the difference between these means. In total, we identified 391/604 oncogenes that have significant promoter-enhancer loops in both A549 and B2B cells, among them 130 (~33%) exhibited increased promoter-enhancer looping events in A549 cancer cell line. These findings indicate that AP-1-mediated promoter looping could be a prevalent mechanism in NSCLC.

### AP-1 appears to control oncogenic expression in NSCLC

**Fig. 4:**
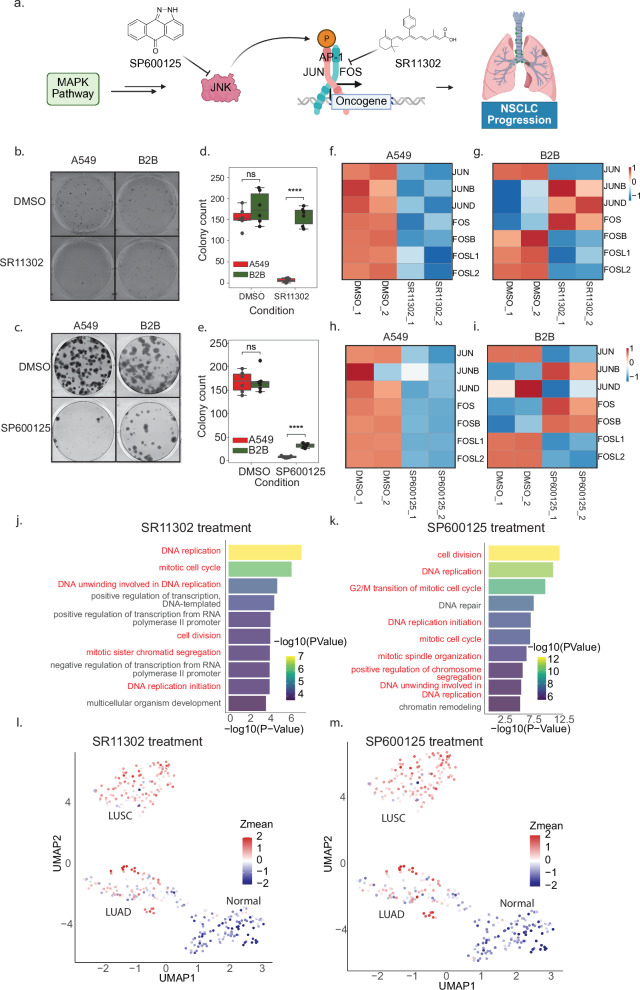
AP-1 inhibitors suppress the transcription of oncogenes. **a** Schematic workflow of inhibition of AP-1 by SR11302 or SP600125. **b**, **c** Colony formation assay (CFA) of A549 and B2B cells treated with DMSO or with 10 μM of AP-1 inhibitor SR11302 (**b**) or 50 μM of JNK inhibitor SP600125. **d**, **e** Box plot illustrating the quantitative analysis of CFA across six biological replicates. A two-tailed *t*-test was performed to assess the statistical significance between the two cell lines (****p value < 0.0001; ns non-significant). Heatmaps displaying Z-score visualization of transcription levels of AP-1 family transcription factors in A549 cells treated with DMSO versus SR11302 (**f**), and in B2B cells (**g**). Heatmaps displaying Z-score visualization of transcription levels of AP-1 family transcription factors in A549 cells treated with DMSO versus SR11302 (**h**), and in B2B cells (**i**). Bar charts showcasing the top 10 Gene Ontology terms of uniquely down-regulated genes in A549 cells following SP11302 treatment (**j**) or SP600125 treatment (**k**). UMAP projection displaying the average value of the z-score, concentrating on genes that are uniquely down-regulated in A549 cells following SR11302 treatment (**l**) or SP600125 treatment (**m**).

Previous studies have demonstrated that activation of AP-1 transcription factors is mediated through phosphorylation of Jun family members by the Jun N-terminal Kinase (JNK) family within the MAPK signaling pathway [9]. To investigate whether AP-1 contributes to oncogenic transcription in NSCLC, we treated A549 and B2B cell lines with the AP-1 inhibitor SR11302 [50, 51] and the JNK inhibitor SP600125 [52] (Fig. 4a). Colony formation assays revealed that 10 µM SR11302 significantly inhibited the growth of A549 cells, with little effect on B2B cells (Figs. 4b, d and S4a). Similarly, treatment with 50 µM SP600125 reduced colony formation in both cell lines, with a more pronounced inhibitory effect in A549 cells (Figs. c, e4 and S4b). These findings suggest that AP-1 plays a cancer-specific role in supporting NSCLC cell growth. To assess the transcriptional consequences of AP-1 inhibition, we performed RNA-seq after 72 h of SR11302 or SP600125 treatment. Interestingly, in A549 cells, all seven major AP-1 subunits were downregulated, whereas in B2B cells, the response was variable, with both upregulation and downregulation observed (Fig. 4f–i). We further identified 960 and 1948 uniquely downregulated genes (including 34 and 71 oncogenes) in A549 cells following SR11302 and SP600125 treatment, respectively, compared to 1069 and 1526 in B2B cells (including 86 and 60 oncogenes) (Fig. S4c, d). Among these two different chemical treatments, we have identified 418 genes that are commonly downregulated specifically in A549 cells (Fig. S4e). Subsequent Gene Ontology (GO) analysis revealed that genes downregulated in A549 were enriched in pathways related to DNA replication and cell cycle, while downregulated genes in B2B were associated with cell adhesion and wound healing (Figs. 4j, k and S4f, g), indicating distinct transcriptional programs affected by AP-1 inhibition in lung cancer versus normal cells. To determine whether these A549-specific AP-1 target genes are relevant in NSCLC patients, we performed UMAP projections using average Z-scores of genes uniquely downregulated in A549 cells following inhibitor treatment. These projections showed that A549-inhibited genes are upregulated in NSCLC samples, especially in LUSC patients (Fig. 4l, m). Consistent with this, Gene Set Enrichment Analysis (GSEA) [53] confirmed transcriptional upregulation of these genes in NSCLC patient cohorts (Fig. S4h, i). Together, these results indicate that AP-1 activates a distinct set of oncogenic genes in NSCLC, particularly in the LUSC subtype, and that pharmacological inhibition of AP-1 suppresses this transcriptional program.

### AP-1 mediates oncogenic expression in NSCLC through promoter-enhancer looping

While most AP-1 family transcription factor genes are recognized as oncogenes, recent studies suggest that a significant proportion of oncogene overexpression in cancer arises from non-genetic mechanisms such as extrachromosomal DNA (ecDNA) amplification [54–56]. To determine whether AP-1 upregulation in NSCLC patients is attributable to genetic alterations, we analyzed gene-level CNV and simple somatic mutation (SSM) data from TCGA-LUAD and TCGA-LUSC cohorts. In contrast to highly amplified oncogenes such as *EGFR* [44], we found no significant CNV increase across most AP-1 subunits, except for modest amplification of *FOSB* in LUSC and *FOSL2* in both LUAD and LUSC (Fig. S5a, b). We also examined CNV and SSM patterns in patients with the highest AP-1 expression (top 25%, Q4 group) and found no significant differences compared to the overall NSCLC cohort (Fig. S5c, d). Furthermore, SSMs in AP-1 subunits were infrequent, particularly compared to recurrently mutated genes like *KRAS*, *EGFR*, and *BRAF* (Fig. S5e–h). These data support the hypothesis that AP-1 upregulation in NSCLC, especially in LUSC, is predominantly driven by non-genetic mechanisms, likely epigenomic in nature.

**Fig. 5:**
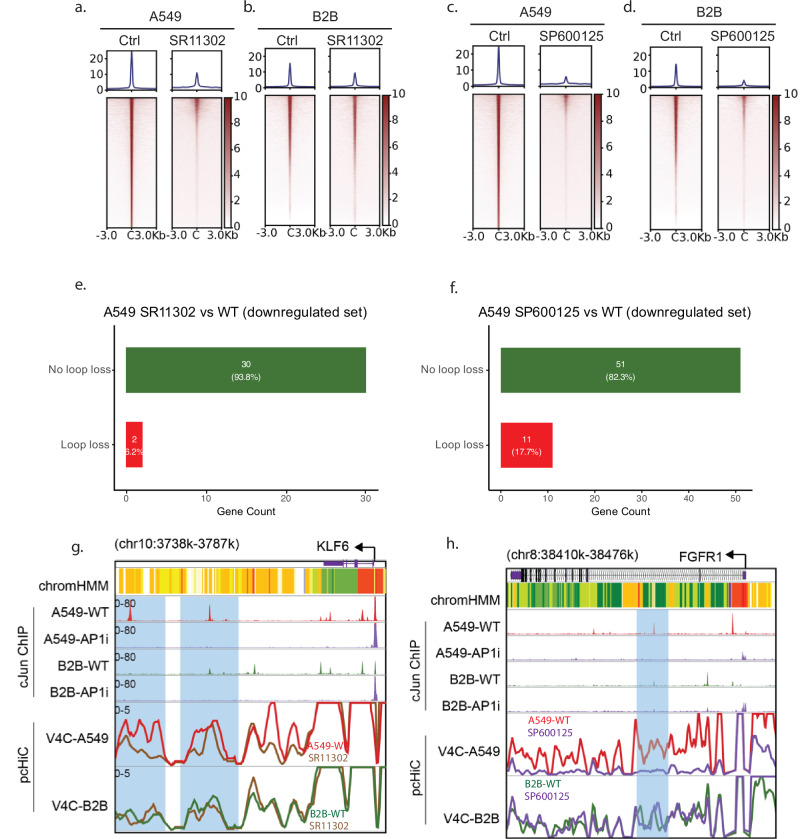
AP-1 inhibition suppresses promoter-enhancer looping. Heatmaps and histogram profiles show the distribution of *cJun* ChIP-Seq in A549 (**a**) and B2B (**b**) cells before (left) and after 72 h of treatment with 10 µM SR11302 (right). Heatmaps and histogram profiles show the distribution of *cJun* ChIP-Seq in A549 (**c**) and B2B (**d**) cells before (left) and after 72 h of treatment with 50 µM SP600125 (right). Bar plot showing the number of downregulated oncogenes exhibiting decreased (red) versus not decreased (green) promoter-enhancer looping events in A549 cancer cells compared to B2B cells following SR11302 (**e**) or SP600125 (**f**) treatment. **g** Browser track visualization of *KLF6*. For Virtual 4 C tracks, wild-type conditions in A549 cells are colored in red, B2B cells in green, and cells treated with SR11302 for 72 h are colored in brown. Significantly altered promoter-enhancer looping structures are highlighted in blue regions. **h** Browser track visualization of *FGFR1*. For Virtual 4 C tracks, wild-type conditions in A549 cells are colored in red, B2B cells in green, and cells treated with SP600125 for 72 h are colored in purple. Significantly altered promoter-enhancer looping structures are highlighted in blue regions.

To test this hypothesis, we performed *cJun* ChIP-seq following AP-1 inhibition via SR11302 or SP600125. Both treatments led to global reductions in c-Jun chromatin binding in A549 and B2B cells, with stronger effects observed in A549 cells (Fig. 5a–d). We next assessed the impact of AP-1 inhibition on chromatin architecture by performing capture Hi-C following SR11302 treatment. In A549 cancer cells, we identified 2 oncogenes with reduced promoter-enhancer looping upon AP-1 inhibition following SR11302 treatment (Fig. 5e), and 11 oncogenes following SP600125 treatment (Fig. 5f). For example, *KLF6*, a transcription factor known to interact with c-Jun and suppress its oncogenic activity [57]. pcHiC analysis revealed two enhancers ~40–50 kb downstream of the *KLF6* promoter that showed active enhancer signatures (EnhA, ChromHMM) and c-Jun binding under basal conditions. These interactions and c-Jun occupancy were reduced following SR11302 treatment (Fig. 5g). Similarly, JNK inhibition via SP600125 resulted in diminished transcription and reduced chromatin looping at the *FGFR1 locus*—another known AP-1 target [58]—specifically in A549 cells (Fig. 5h). pcHiC analysis revealed a global decrease of promoter looping structures at the *FGFR1* locus in A549 cells, including an enhancer within the gene body region (highlighted in blue) that colocalized with *cJun* ChIP signals (Fig. 5h). These *cJun* signals were diminished following JNK inhibition (Fig. 5h). In contrast, B2B cells largely maintained the same chromatin looping structures after JNK inhibition, indicating that this mechanism is selectively engaged in cancer cells. Together, these findings demonstrate that AP-1 mediates oncogenic transcription in NSCLC by promoting promoter-enhancer looping, and that this regulation is largely driven by epigenomic mechanisms rather than genetic alterations in AP-1 itself.

## Discussion

A major challenge in cancer research lies in integrating large-scale genomic datasets, such as those from TCGA, with regulatory information, including transcription factor binding and chromatin architecture. This integration is essential for elucidating the mechanisms driving oncogenic transcription. In this study, we established a paired model system comprising the NSCLC cell line A549 and the non-cancerous bronchial epithelial cell line B2B to systematically investigate transcriptomic, epigenomic, and 3D genomic alterations associated with lung cancer progression. A549 cells, though originally derived from lung adenocarcinoma and exhibiting features of alveolar type II (AT2) epithelial cells, display a hybrid phenotype characterized by high tumorigenic potential, rapid proliferation, oncogenic chromatin accessibility, and expression of basal/squamous markers such as TP63, along with molecular and epigenomic features resembling squamous cell carcinoma [59]. This phenotypic plasticity makes A549 a suitable model for interrogating LUSC-associated transcriptional programs. In contrast, BEAS-2B cells are SV40-immortalized bronchial epithelial cells with basal-like or squamous differentiation potential and lack tumorigenicity [60]. Using a comprehensive multi-omic strategy including pcHiC, ATAC-Seq, ChIP-Seq, and RNA-Seq, we identified significant alterations in gene expression and epigenomic landscape that underpin oncogenic processes in NSCLC. Our integrated analysis confirmed that this model cell line system recapitulates key oncogenic transcriptional patterns observed in TCGA NSCLC samples (Figs. 1d and S1h). Since the TCGA LUAD and LUSC ATAC-seq datasets are tumor-only and lack matched normal controls, we leveraged differential ATAC-seq peaks from A549 and B2B cells to benchmark chromatin accessibility. The ATAC-seq profiles from TCGA tumor samples showed significantly greater overlap with A549-enriched regions than with B2B-enriched regions, indicating that our model system more accurately reflects the chromatin accessibility landscape of NSCLC patients. Further integration with ChIP-seq data for active promoter (Pol II, H3K4me3), enhancer (Med1, H3K27ac), and insulator (Rad21, CTCF) marks revealed a global gain of active regulatory elements and a loss of insulators in NSCLC.

To identify candidate transcription factors responsible for these oncogenic alterations, we conducted motif analysis of enhancers associated with differentially expressed oncogenes. We merged significantly differentially expressed oncogene-looped enhancers with overlapping open chromatin regions and conducted motif analysis. Our analysis revealed significant differences in motif enrichment between the A549 and B2B cell lines. Notably, the insulator hallmark *CTCF* was uniquely highlighted in A549 cells, suggesting potential chromatin structural changes in lung cancer cells [61]. More importantly, nine out of the top ten motifs identified in A549 cells belonged to the AP-1 transcription factor family, underscoring AP-1’s pivotal role in driving oncogenic transcriptional programs in lung cancer. Previous research has indicated that multiple feedback loops compensate for different AP-1 subunits, making it difficult to detect significant differences when comparing each subunit individually. We thus calculated the mean transcription intensity across all AP-1 subunits and used this composite measure to compare survival rates among different patient groups. The significant correlation between average AP-1 transcription levels and the survival rates of patients with LUSC suggests that abnormal AP-1 activation is prevalent in LUSC.

Using pcHiC, we observed a pervasive gain of promoter–enhancer loops at oncogene loci in A549 versus B2B cells. Spearman’s correlation confirmed that these loops, particularly promoter-enhancer loops, are closely associated with transcriptional output. Strikingly, AP-1 occupancy was significantly enriched at both anchors of these loops, and virtual 4 C traces verified AP-1-dependent enhancer–promoter contacts at key oncogenes such as EGFR and SRC. Although AP-1 is known to recruit SWI/SNF to open chromatin at enhancers [62, 63], our data reveal a separate, structural role: AP-1 dimers physically “bridge” distal enhancers to their target promoters, boosting 3D contacts and driving transcription, thereby functionally coupling enhancer engagement to oncogene activation. To our knowledge, this is the first demonstration in NSCLC that AP-1 directly tethers enhancers to promoters, functionally coupling chromatin looping with oncogene activation.

Interestingly, genomic analysis of TCGA cohorts revealed no significant CNVs or somatic mutations in AP-1 subunit genes, suggesting a non-genetic mechanism of AP-1 activation. We hypothesized that AP-1 is activated via JNK-mediated phosphorylation within the MAPK pathway. However, targeting transcription factors in cancer therapy presents unique challenges due to their pivotal roles in regulating multiple genes and complex protein-protein interactions [7]. Specifically, inhibiting AP-1 is difficult because of the positive and negative feedback loops that exist among different AP-1 subunit genes [12]. We attempted to inhibit the AP-1 transcription factor using siRNA or shRNA technologies; however, due to compensatory mechanisms among AP-1 subunits, this approach was unsuccessful. Nevertheless, small molecule inhibition using SR11302 (direct AP-1 inhibitor) and SP600125 (JNK pathway inhibitor) effectively suppressed AP-1 activity in A549 cells. Both our RNA-seq assays and ChIP-Seq assays demonstrate effective AP-1 inhibition in cancer cells. Moreover, our study shows that inhibition of AP-1 can effectively prevent oncogenic transcription in A549 cells, whereas their impact on B2B cells was notably different. GO analysis of downregulated genes revealed distinct biological processes between A549 (e.g., cell cycle, DNA replication) and B2B (e.g., adhesion, wound healing), emphasizing the cancer-specific role of AP-1. Additionally, pcHiC analysis post-inhibition revealed loss of promoter-enhancer loops at several AP-1 target genes, correlating with reduced transcription. In future studies, higher-resolution chromatin conformation capture techniques such as H3K27ac HiChIP, which enrich specifically for active promoter–enhancer contacts, may further enhance the detection of loop disruptions associated with transcriptional repression upon AP-1 inhibition.

In addition to the canonical MAPK-JNK signaling cascade, transcription factor crosstalk may also contribute to AP-1 activation. Previous studies have shown that *YAP1*, a key oncogenic effector of the Hippo pathway, can cooperate with AP-1 family members such as *FOS* to drive tumor-promoting gene expression [64]. More importantly, Yu et al. has shown that *YAP1* can promote proliferation, metastasis, and epithelial–mesenchymal transition (EMT) in NSCLC through cooperation with TEAD transcription factors [65]. Thus, YAP1–AP-1 crosstalk may represent an alternative mechanism of AP-1 activation in NSCLC. Such transcriptional crosstalk warrants further investigation as a potential avenue for combinatorial therapeutic strategies.

Collectively, our findings demonstrate that AP-1 is aberrantly activated in a subset of NSCLC, where it promotes oncogenic transcription via promoter-enhancer looping. Our findings underscore AP-1’s unique role in remodeling chromatin topology and driving malignant gene expression in NSCLC. These findings suggest that targeting AP-1, particularly through more selective downstream inhibitors, could represent a promising therapeutic avenue for NSCLC. Given AP-1’s role in modulating cancer-specific regulatory networks, future efforts may focus on refining AP-1-targeting strategies with improved specificity and minimized off-target effects to enhance clinical utility.

## Supplementary information

Supplementary Table 1

Supplementary Table 2

Supplementary Table 3

Supplementary Table 4

Supplementary Table 5

Supplementary Table 6

Supplementary Table 7

Supplementary Table 8

Supplementary Table 9

Supplementary Figures

## Data Availability

The accession number for the sequencing data are GSE267596, GSE267597, GSE267598, and GSE267599.
