# Supplementary material for: AP-1 promotes oncogenic transcription in lung cancer cells by bridging promoter-enhancer interactions
Source: Cancer Gene Ther. 2025 Dec 3;33(3):301–13. doi: 10.1038/s41417-025-00974-w (PMC13109059; doi:10.1038/s41417-025-00974-w)
Supplement: Supplementary file 10 — Supplementary Figures [file 41417_2025_974_MOESM10_ESM.pdf]

AP-1 Promotes Oncogenic Transcription in Lung Cancer Cells by Bridging Promoter-Enhancer Interactions

Supplementary Figures

Figure S1

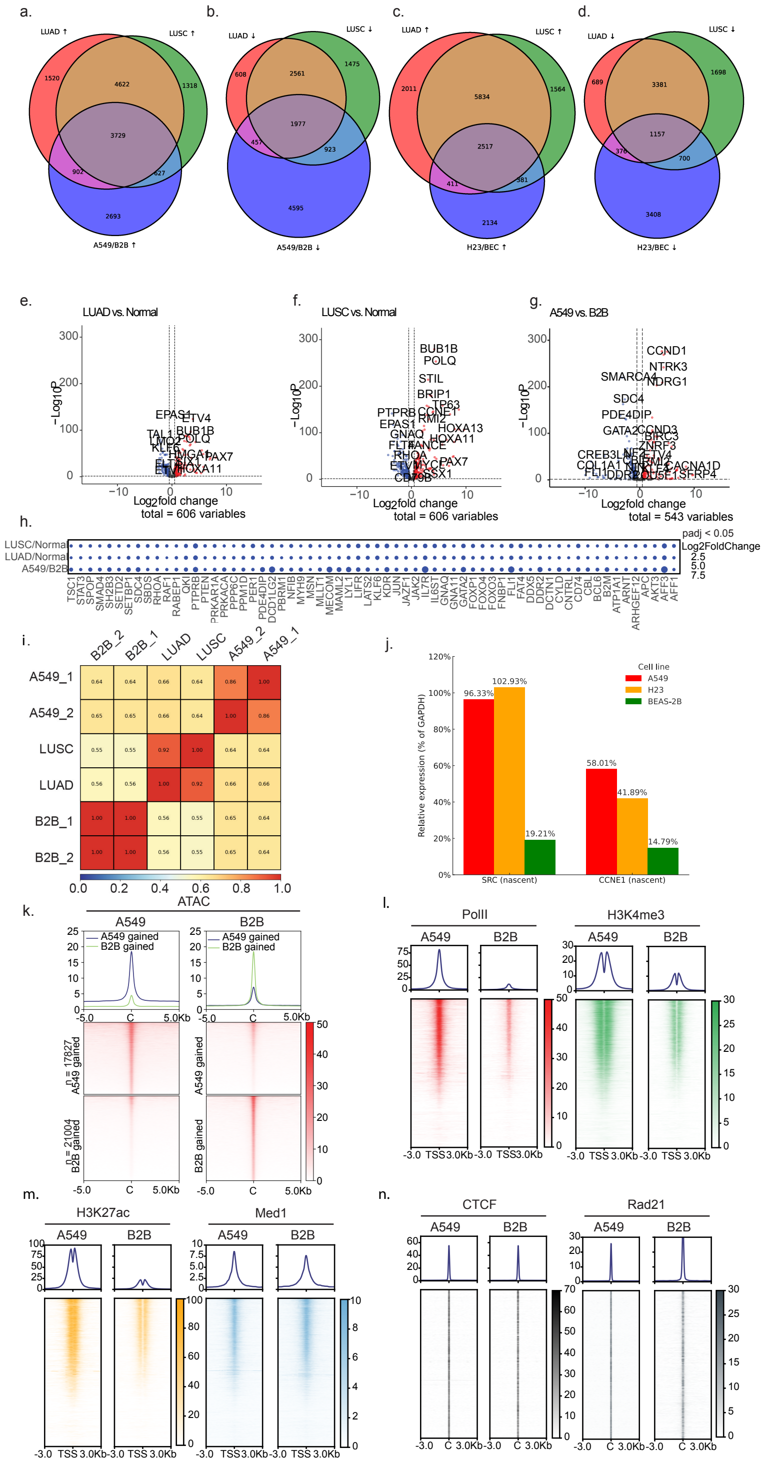

Figure S1. Transcriptomic and epigenomic alterations in NSCLC.

(a-d) Venn-diagrams showing the overlap of global differentially expressed genes (adjusted p < 0.05, |Log2FC| >= 0.5) in three pairwise comparisons: (a). ç A549 versus B2B. (b). Downregulated genes in LUAD versus normal lung tissue, LUSC versus normal lung tissue, and A549 versus B2B. (c). Upregulated genes in LUAD versus normal lung tissue, LUSC versus normal lung tissue, and H23 versus BEC. (d). Downregulated genes in LUAD versus normal lung tissue, LUSC versus normal lung tissue, and H23 versus BEC.

(e-g). Volcano plot depicting differentially expressed oncogenes in LUAD compared to normal tissue (e). LUSC compared to normal tissue (f), and A549 relative to B2B (g). Significantly upregulated genes (Log2FC >= 0.5) are highlighted in red, while downregulated genes (Log2FC <= -0.5) are marked in blue.

(h). Bubble plot depicting oncogenes significantly downregulated in LUAD, LUSC patient samples, and model cell lines. Significance was determined using DESeq2 with adjusted p-values (Padj < 0.05) after multiple testing correction.

(i). Heatmap displays Pearson's correlation analysis of ATAC-Seq data from combined TCGA-LUAD (n = 44) and TCGA-LUSC (n = 32) cohorts, compared with A549 (n = 3) and B2B (n = 2) cells.

(j). RT-qPCR relative expression of SRC and CCNE1 in A549, H23, and BEAS-2B cells. Expression values were normalized to GAPDH and calculated using the comparative Ct (2^−ΔΔCt) method. Each condition was assayed in three technical replicates, and measurements were normalized to the mean value of those replicates.

(k). Heatmap and histogram profiles of ATAC-Seq signals in A549 and B2B cells clustered by differential binding peak loci in each cell line.

(l). Heatmaps and histogram profiles of ChIP-Seq signals targeting Pol II (colored in red) and H3K4me3 (colored in green) in A549 and B2B cells. The transcription start site (TSS) of each gene, covering the central region and extending ±3kb, is plotted and ranked according to its RPKM value in each cell line.

(m). Heatmaps and histogram profiles of ChIP-Seq signals targeting H3K27ac (colored in yellow) and Med1 (colored in blue) in A549 and B2B cells. The TSS of each gene, covering the central region and extending ±3kb, is plotted and ranked according to its RPKM value in each cell line.

(n). Heatmaps and histogram profiles of ChIP-Seq signals targeting CTCF (colored in black) and Rad21 (colored in gray) in A549 and B2B cells are displayed. For each A549 cell line, the peak sites of CTCF and Rad21 in the A549 cell line are plotted in the central region, extending ±3kb for each cell line. TSS: Transcription start site. C: Peak centers.

Figure s2

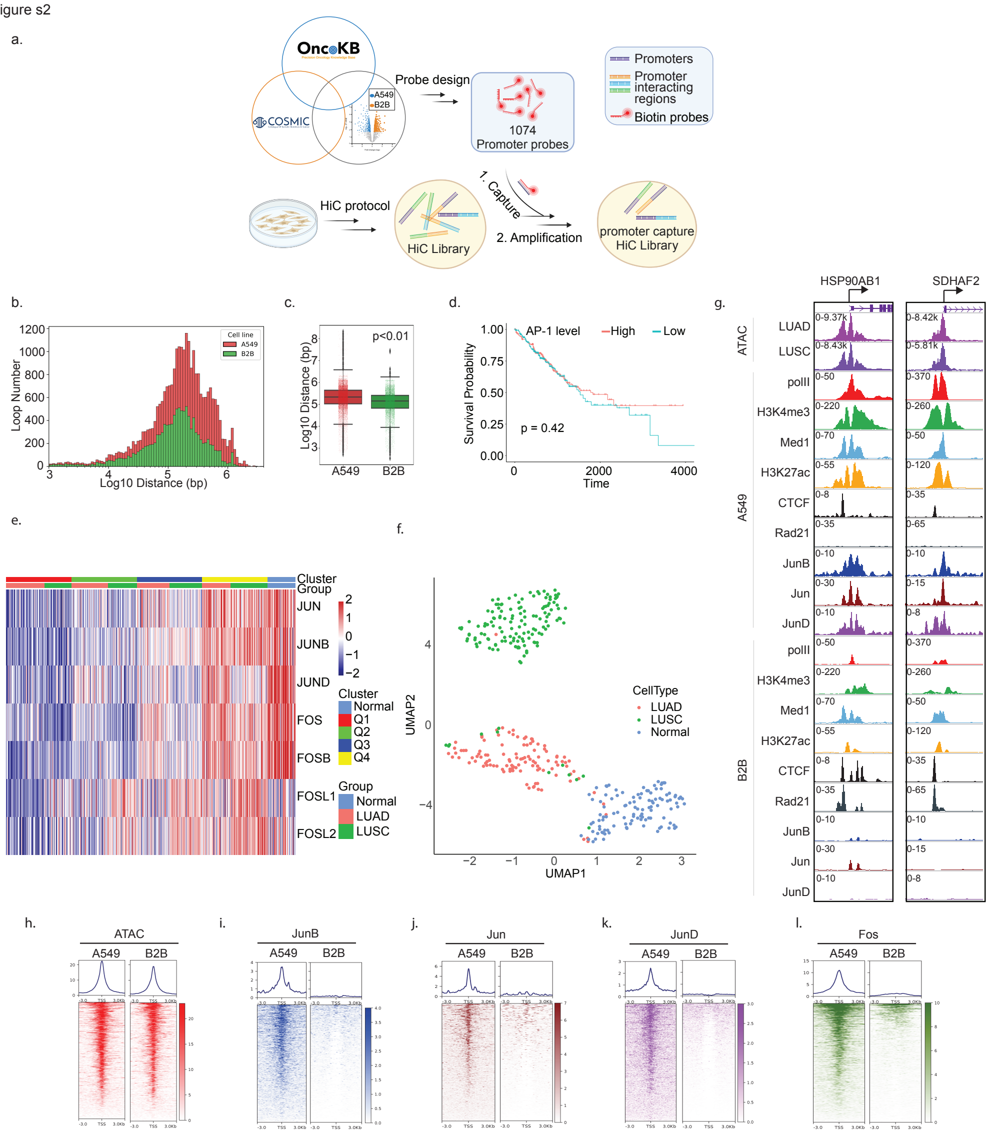

Figure S2. Transcription factor AP-1 is aberrantly activated in NSCLC.

(a). Schematic representation of the workflow for pcHiC probe design targeting oncogenes and highly transcribed genes in A549 and B2B cells.

(b). Distribution of distance and interaction intensity between promoters and promoter-interacting regions (PIRs) for the selected gene set in A549 and B2B cells.

(c). Box plot of promoter looping distances between A549 and B2B cells.

(d). Kaplan-Meier survival curves depicting patients with varying levels of AP-1 expression intensity in TCGA-LUAD cohort.

(e). Heatmap illustrates the sub-clustering of TCGA-LUAD and TCGA-LUSC cancer samples into four quantiles based on their AP-1 transcriptional intensity.

(f). UMAP projection of quantile 4 (Q4) from (e) with normal samples tissues.

(g). Browser track representation displaying Pol II, H3K4me3, Med1, H3K27ac, CTCF, Rad21, JunB, Jun, and JunD ChIP-seq data from A549 and B2B cell lines, focus on TSSs of two upregulated oncogenes, HSP90AB1 and SDHAF2. Each track was normalized by counts per million (CPM) between A549 and B2B cells.

(h-l). Heatmaps and histogram profiles demonstrate the distribution of ATAC (h) and the AP-1 family transcription factors binding at oncogene promoters for JunB (i), Jun (j), JunD (k) and Fos (l) in A549 and B2B cells.

Figure s3

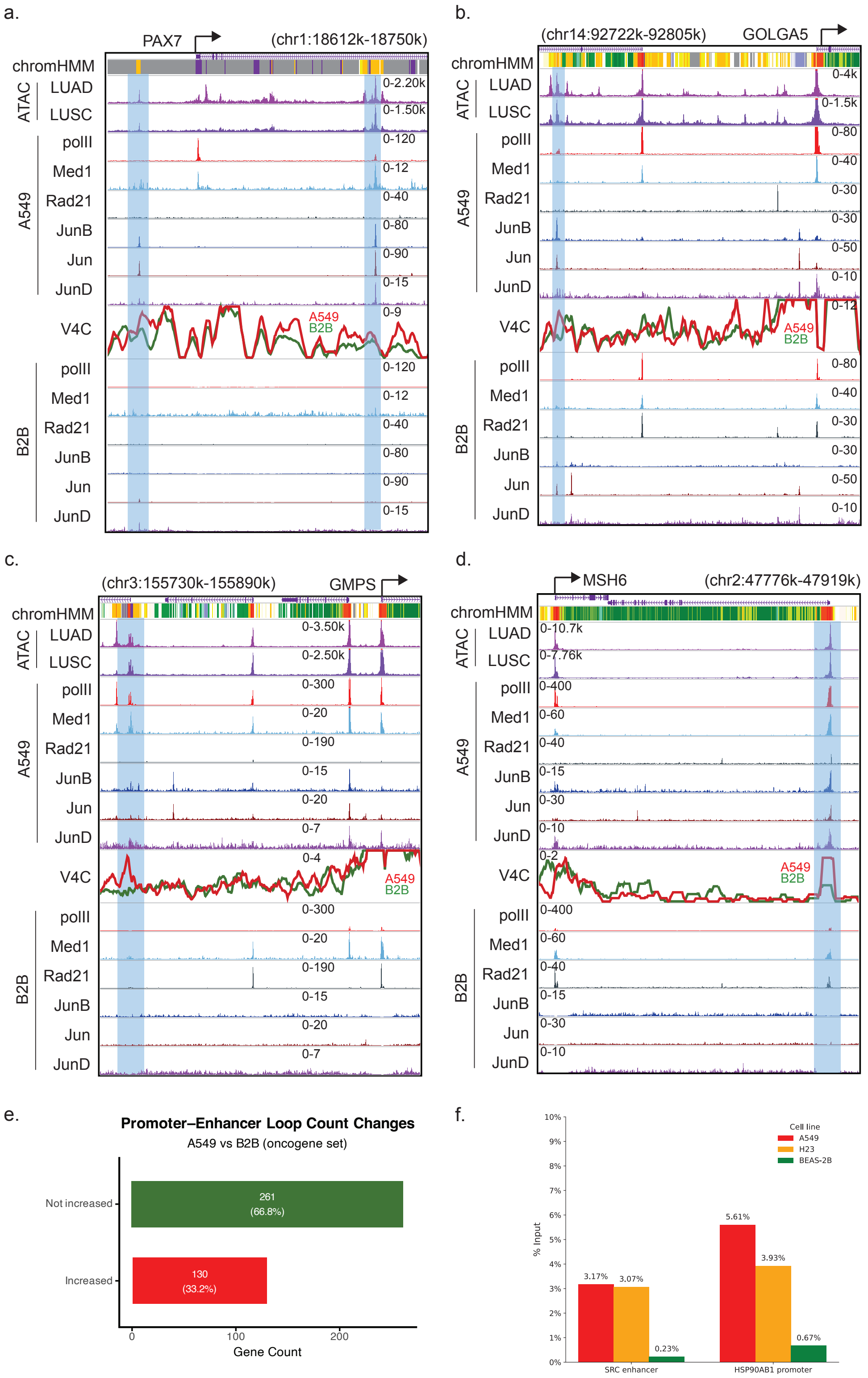

Figure S3. Browser track examples illustrating upregulated oncogenes with AP-1 driven promoter loops.

(a-d). Browser track showcases gene PAX7(a), GOLGA5(b), GMPS(c), and MSH6 (d)

(e). Bar plot showing the number of oncogenes with increased (red) versus not increased (green) promoter-enhancer looping events in A549 cancer cells compared to B2B cells.

(f). ChIP-qPCR analysis of Fos occupancy at the SRC distal enhancer region from Figure 3h and HSP90ab1 promoter region from Figure S2g in A549, H23 and B2B cells. Enrichment levels were quantified using the % input method and are presented as mean values across three technical replicates.

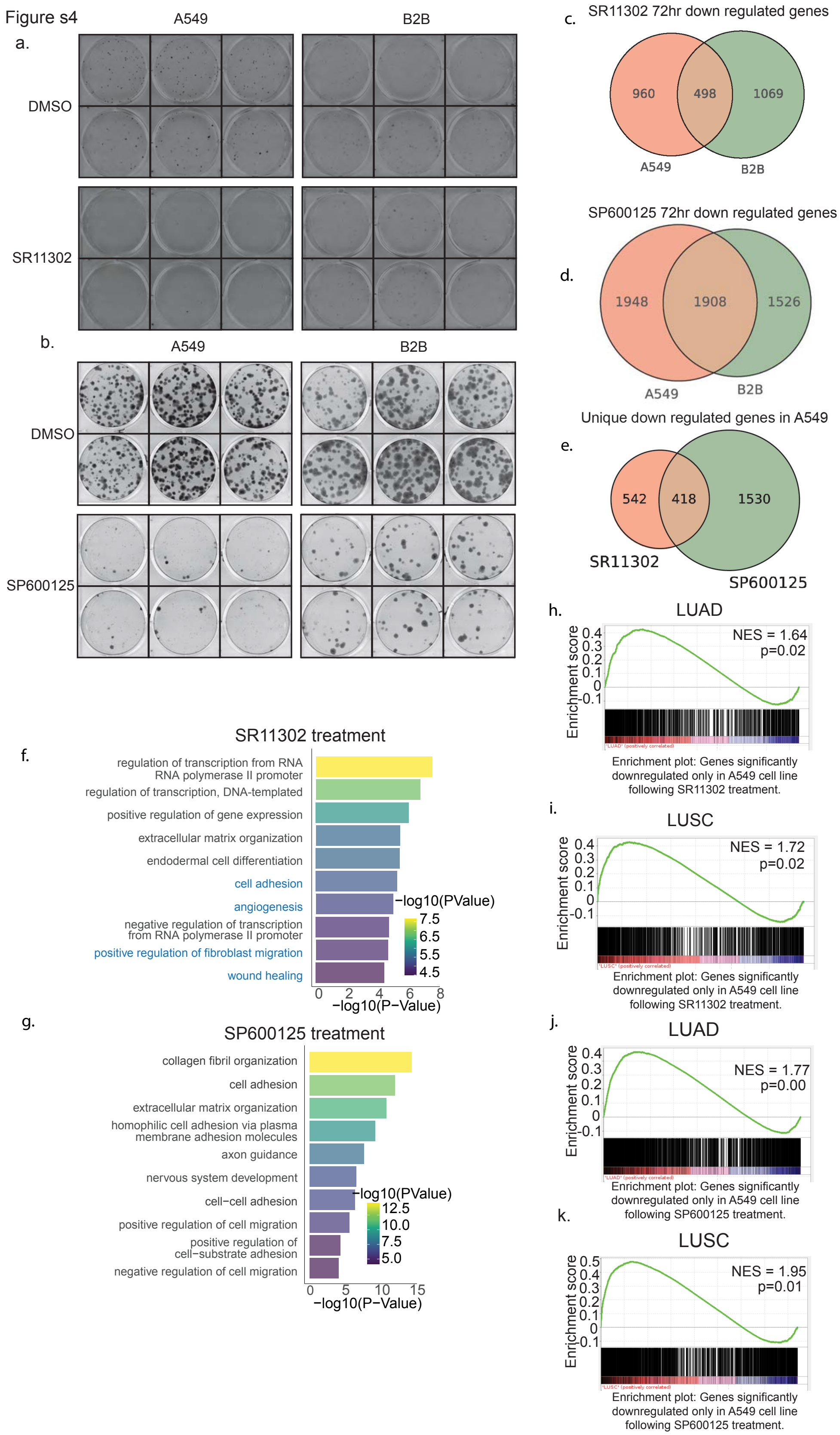
Figure s4
a.
A549
B2B
DMSO
SR11302
b.
A549
B2B
DMSO
SP600125
c.
SR11302 72hr down regulated genes
960
498
1069
A549
B2B
d.
SP600125 72hr down regulated genes
1948
1908
1526
A549
B2B
e.
Unique down regulated genes in A549
542
418
1530
SR11302
SP600125
f.
SR11302 treatment
regulation of transcription from RNA RNA polymerase II promoter
regulation of transcription, DNA-templated
positive regulation of gene expression
extracellular matrix organization
endodermal cell differentiation
cell adhesion
angiogenesis
negative regulation of transcription from RNA polymerase II promoter
positive regulation of fibroblast migration
wound healing
−log10(PValue)
7.5
6.5
5.5
4.5
0 2 4 6 8
−log10(P−Value)
g.
SP600125 treatment
collagen fibril organization
cell adhesion
extracellular matrix organization
homophilic cell adhesion via plasma membrane adhesion molecules
axon guidance
nervous system development
cell−cell adhesion
positive regulation of cell migration
positive regulation of cell−substrate adhesion
negative regulation of cell migration
−log10(PValue)
12.5
10.0
7.5
5.0
0 5 10 15
−log10(P−Value)
h.
LUAD
NES = 1.64
p=0.02
Enrichment score
Enrichment plot: Genes significantly downregulated only in A549 cell line following SR11302 treatment.
i.
LUSC
NES = 1.72
p=0.02
Enrichment score
Enrichment plot: Genes significantly downregulated only in A549 cell line following SR11302 treatment.
j.
LUAD
NES = 1.77
p=0.00
Enrichment score
Enrichment plot: Genes significantly downregulated only in A549 cell line following SP600125 treatment.
k.
LUSC
NES = 1.95
p=0.01
Enrichment score
Enrichment plot: Genes significantly downregulated only in A549 cell line following SP600125 treatment.

Figure S4. AP-1 inhibitors suppress the transcription of oncogenes.

(a-b). Colony Formation Assay (CFA) of A549 and B2B cells treated with DMSO or with 10uM of AP-1 inhibitor SR11302 (a) or 50uM of JNK inhibitor SP600125.

(c-d). Venn diagram illustrating the overlap of significantly downregulated genes following SR11302 treatment (c) or SP600125 treatment (d) (Log2FC < 0.5) between A549 and B2B cells.

(e). Venn diagram illustrating the overlap of significantly downregulated genes specifically in A549 cells genes following SR11302 treatment or SP600125 treatment (Log2FC < 0.5) between A549 and B2B cells.

(f-g). Bar charts showcasing the top 10 Gene Ontology terms of uniquely down-regulated genes in B2B cells following SP11302 treatment (e) or SP600125 treatment (f).

(h-i). Gene Set Enrichment Analysis (GSEA) of gene sets uniquely down-regulated in the A549 cell line, as applied to TCGA-LUAD (g) and TCGA-LUSC (h) cohorts. Each gene is represented as a bar at the bottom, with genes positively correlated and sorted to the left. The enrichment profile is displayed in green.

(j-k). GSEA focuses on genes uniquely down-regulated in A549 cells after SP600125 treatment, applied to TCGA-LUAD (i) and TCGA-LUSC (j) cohorts. Each gene is represented as a bar at the bottom, with genes positively correlated and sorted to the left. The enrichment profile is displayed in green.

Figure s5

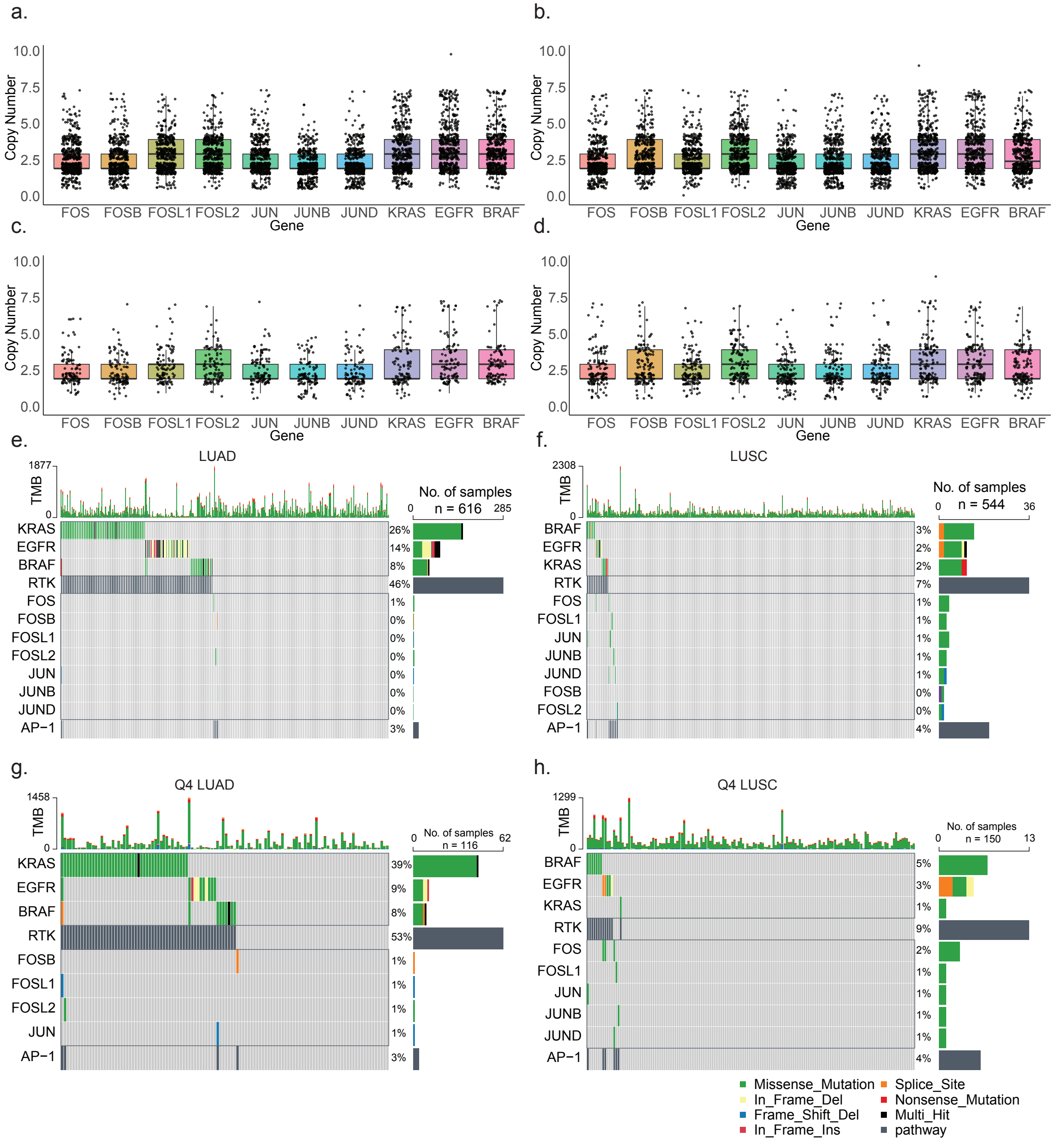

Figure S5. Activation of AP-1 in NSCLC is not driven by genetic mutations in AP-1 genes. (a-d). Boxplots comparing the copy number of AP-1 family genes with those of classical RTK pathway genes KRAS, EGFR, and BRAF across different groups: the total LUAD cohort (a), the total LUSC cohort (b), Q4 LUAD patients (c), and Q4 LUSC patients (d).

(e-h). Oncoplot illustrating somatic mutations in AP-1 family genes with those of classical RTK pathway genes KRAS, EGFR, and BRAF across different groups: the total LUAD cohort (e), the total LUSC cohort (f), Q4 LUAD patients (g), and Q4 LUSC patients (h).
